# Supplementary material for: A Community-Based Approach to Integrating Socio, Cultural and Environmental Contexts in the Development of a Food Database for Indigenous and Rural Populations: The Case of the Batwa and Bakiga in South-Western Uganda
Source: Nutrients. 2021 Oct 3;13(10):3503. doi: 10.3390/nu13103503 (PMC8537349; doi:10.3390/nu13103503)
Supplement: Supplementary file 1 [file nutrients-13-03503-s001.zip › nutrients-1367702-supplementary.pdf]

Supplementary Materials

Supplementary Material 1

Template for the food list data collection in shops and markets.

MARKET ☐ SHOP ☐

PRODUCT TYPE	BRAND (if any)	COST	WEIGHT (if packaged food)	Seasonal availability	Comments

AREA: Please specify the area of the shop/market visited.

Supplementary Material 2

Guide topics for the focus group discussions.

a) Which foods do you usually buy from the shops and markets?

b) Do you eat packaged food from the market or from the shops? What foods?

c) Which drinks and snacks do you consume?

d) What foods do you produce and eat (e.g. from you garden, animals you raise)?

e) Do you eat any food from the forest, any roots or anything you pick up from trees? If yes, which one(s)?

f) Do you sometimes receive food as a gift? Which foods do you receive?

g) What do you usually eat during holidays or during any celebrations? Is this food different from usual?

f) Is there any type of food that you rarely eat (or used to eat) and has not been mentioned before?

h) Can you please tell how each food (mentioned by you) is usually cooked among your community?

i) Can you please tell how each food (mentioned by you) is usually processed among your community?

j) Can you please tell how each food (mentioned by you) is usually stored among your community?

k) Do children, women and men eat different amounts of food? How much?

l) Are there differences in the availability of this specific food during the dry and rainy season? If yes, can you please tell me the type of foods that you find in the dry and wet season?

m) Did you see a change of the availability of this food over time? How do you adapt to these changes? Have you changed/substituted the food you eat?

Supplementary Material 3

Probe questions used for the dietary survey.

a) How many meals did you (or your child) have yesterday?

Is this your (or your child's) usual number of meals?

b) What did you (or your child) eat yesterday in the morning? Can you describe what did you eat? How it was cooked? How can we measure the food that you have eaten (which tools could help to measure each food?)

Is this your (or your child's) usual meal?

c) What did you (or your child) eat yesterday in the afternoon? Can you describe what did you eat? How it was cooked? How can we measure the food that you have eaten (which tools could help to measure each food?)

Is this your (or your child's) usual meal?

d) What did you (or your child) eat yesterday in the evening? Can you describe what did you eat? How it was cooked? How can we measure the food that you have eaten (which tools could help to measure each food?)

Is this your (or your child's) usual meal?

e) Snacks? Other eating occasions? Drinks? Is this a usual day?
If no, why not?

Paper form

Meal/time	***Food item***	***Ingredients***	***Preparation method***	***Brand name (commercial product)***	***Tools used to measure each food***	***Comments***
**Breakfast**						
**Any snacks?**						
**Lunch**						
**Any snacks?**						
**Dinner**						
**Before bed/ bedtime snack**						
